# Annotations

**Published:** 1901-09-21

**Authors:** 


### Annotations.

### The Cell and the Molecule.

Among the more interesting addresses read at the meeting of the British Association at Glasgow was that by Professor McKendrick on molecular physiology. He entered upon the very curious, and from a physiological point of view, the very interesting question as to the number of organic molecules which can be contained in the smallest particle of living matter, and showed the practical nature of the inquiry by discussing whether there can be in the ovum, for example, a sufficient number of molecules to account for the facts of hereditary transmission. In the course of his address he pointed out that much as we know about the broader physiology of organs, when we come to discuss the phenomena of the living tissues we find ourselves in difficulties from the fact that we still are but imperfectly acquainted with the changes which take place in a living cell, changes upon which the phenomena of life depend. By various means we are able to analyse the cell-substance into a variety of matters, but we obtain little information as to how these proximate constituents are built up into the living substances of the cell. Yet it is on this that everything hinges, for if function depends upon structure the question arises how can all the different tendencies of hereditary influence be packed away in the small space of a germ cell? Here we are face to face with a serious enough difficulty. But Professor Cunningham, when he brings the matter to figures, shows that the difficulty is not as great as some might imagine it to be, for by comparing the estimates which have been made as to the size of an organic molecule with what we know as to the size of the germinal vesicle of the ovum, and the head of the spermatozoid, which is all that is necessary for fecundation, and after allowing for the large amount of water which these bodies contain, he concludes that the fecundated ovum might start on its life with 12,000,000,000,000 organic molecules. Hence he thought that Clark Maxwell's argument that there were too few organic molecules in an ovum to account for the transmission of hereditary peculiarities, did not hold good, for instead of the organic molecules in the germinal vesicle of an ovum numbering something like a million, the fecundated ovum probably contained millions of millions.

### Civilisation and Disease.

One can hardly have a doubt that many of the diseases with which man is afflicted are connected in some more or less vague and mysterious manner with the food he eats. It is the penalty we pay for being guided by intellect rather than instinct. In the course of ages, by dint of the stability of their surroundings, animals develop an instinct in regard to their food which tells them unerringly what to eat and what to avoid. Man, however, is always surrounding himself with new conditions. Looking at the matter from a purely evolutionary standpoint the wonder is that he survives at all. To heat and cold, to tropics and to arctic regions he tries to accustom himself, while as regards diet he takes his chance at all that offers. Meat and vegetables, fruits and nuts, fish and fowl; fresh, preserved, dried in the sun, baked in ovens, boiled down and condensed, sealed up in tins and jars of every description, and in latter years pickled in every deleterious antiseptic that the ingenuity of the chemist can invent. We need hardly wonder then that man is somewhat less hardy than animals which are more perfectly fitted to their more stable environment, and that the complete alteration of all the conditions of man's life which have been suddenly introduced by the invention of the steam engine—by which not only is man carried rapidly to all parts of the world, but the products of all parts of the world are brought to man, and placed without more ado upon his breakfast table—should have had the effect of strewing man's trail with a considerable number of failures losses from consumption, from insanity, from fevers of all kinds, and from cancer. Whatever may be the immediate cause of cancer we can hardly doubt that it has of late years shown a tendency to increase, or that this increase has especially affected the "progressive" nations, those in which all the old habits and associations, especially in regard to locality and food, have been subjected to a violent process of topsy-turveydom. The old natural conditions in the midst of which different men and different races grew up each in his or its own native air have been shaken up, and injurious influences which in former times might have affected villages or small communities, or might by their deleterious effects have mapped out certain districts as unfit for human habitation, have been so widely spread by trucks and steamships as to introduce new and unknown conditions into the lives of thousands in distant parts of the world, conditions against which man, whose intellect has to a large extent destroyed his instinct, has not the natural protection of that "brute instinct" which is the attribute of the animal creation in a state of nature.

### Oh, Those Newspapers!

We hear so much at the present day about the lavish expenditure which the managers of great newspapers are prepared to undertake in the hope of excelling their rivals, that it is a matter of standing wonder to us that these rich and wealthy corporations do not spend an occasional five-pound note in so serving up their contents as to render them acceptable to thinking men of ordinary intelligence. As it is, everyone who is endowed with that reasonable amount of scepticism which is necessary to enable a man to go through life without being tripped up at every turn, takes his newspaper with so many grains of salt that he loses all its flavour. Take the following delightful bit of intelligence with which we were regaled last Friday morning. A physician in America is stated to have demonstrated the use of a fluid invented by himself which he asserts will cure consumption. "In the presence of a dozen members of the medical profession the solution was injected into the veins of five persons in advanced stages of tuberculosis. The results are said to have been *immediately beneficial in each case*." Surely doctors are cheap enough, and a newspaper that can afford to send its special correspondents to the uttermost parts of the earth in search of news paragraphs, might, we should have thought, have kept some man of sense upon the premises to save it from such absurdities as this. If a man had even the remotest idea of the condition of another's inside when he is suffering from an advanced stage of tuberculosis, he would know what nonsense he was talking when he spoke of the results being "immediately beneficial," and if, as of course is probable, he knew absolutely nothing about the subject, why in the name of honesty could he not let it alone. Mere newspaper twaddle! That is the comment heard on every side when anything occurs out of the common now. And can we wonder.

